# Sepsis in burns: lessons learned, challenges remain

**DOI:** 10.1186/s40779-025-00677-1

**Published:** 2025-12-02

**Authors:** Folke Sjöberg, David Greenhalgh, Moustafa Elmasry, Ahmed T. El-Serafi, Ingrid Steinvall

**Affiliations:** 1 Department of Hand Surgery, Plastic Surgery, and Burns, Linköping University, 581 85 Linköping, Sweden; 2 Department of Biomedical and Clinical Sciences, Linköping University, 581 85 Linköping, Sweden; 3 Department of Burns, Shriners Children’s Northern California and Department of Surgery, University of California, Davis, Sacramento, CA 95817-2215 USA; 4 Medical Laboratory Science Department, College of Health Sciences, University of Sharjah, 27272, Sharjah, United Arab Emirates

**Keywords:** Burn injury, Sepsis, Epidemiology, Biomarkers, Artificial intelligence, Antibiotics

Sepsis continues to be a leading cause of adverse outcomes in burn patients [1–6]. Despite advances in resuscitation, surgery, and critical care, septic complications contribute substantially to long-term morbidity and mortality. Epidemiological studies are surprisingly few, especially those including patients with smaller burn sizes, which represent the majority of admissions in high-income settings. In this context, the work of Tedesco et al. [7] is welcome. The study of more than 1400 patients admitted between 2006 and 2025 demonstrates that early post-burn sepsis substantially worsens prognosis in both adults and older adults. The authors also confirm that burn size, age, and inhalation injury remain independent risk factors for sepsis.

The strengths of this study include its large sample size, multicenter setting, and adherence to STROBE guidelines [7]. The inclusion of smaller burns [≥ 5% total body surface area (TBSA)] broadens its relevance [7]. Importantly, the finding that sepsis increases mortality even in smaller burns deserves emphasis [7].

However, as a retrospective analysis, the study is inevitably constrained. Sepsis onset was reported at a median of 10 d [7], which appears later than other recent studies, including a Korean cohort of more than 1300 patients, where the mean onset was only 4 d [6]. This discrepancy highlights how definitions, case mix, and methodology influence apparent timing. Moreover, many retrospective reports, including this one, do not specify whether prophylactic antibiotics were used [4]. Given the well-recognized risk of driving multidrug resistance, the antibiotic strategy is a critical confounder that must be explicitly reported.

The next stage of burn sepsis research should be prospective, with high temporal resolution to capture both sepsis criteria and infection signs dynamically. Harmonization around Sepsis-3 criteria, now increasingly validated in burn cohorts, is essential [2]. Such studies should include structured biomarker sampling (e.g., procalcitonin, C-reactive protein, lactate, cytokine profiles), integrated with advanced computational approaches. Machine learning and artificial intelligence (AI) techniques are well-suited to identify non-linear trajectories and early signatures of sepsis that elude conventional analyses. In parallel, careful documentation of antibiotic protocols is needed to clarify their impact on sepsis incidence and resistance patterns [4]. Ultimately, only prospective, biomarker-rich, and AI-enhanced studies will allow personalized risk stratification and earlier, more effective interventions [2].

In support of this harmonization, several recent studies have directly evaluated the Sepsis-3 criteria in burn populations (Table 1). These include cohorts from Sweden, Canada, and South Korea, which consistently demonstrate that Sepsis-3 definitions, based on a Sequential Organ Failure Assessment (SOFA) score increase ≥ 2, are currently the most robust and reproducible for detecting clinically relevant sepsis in burn patients [4–6]. Across studies, sepsis onset typically occurs within the first week post-injury, highlighting the need for early monitoring and high-frequency data capture.

**Table 1:** Summary of recent studies applying Sepsis-3 criteria in burn patients

Study	Population	Sepsis incidence	Time to onset	Key findings
Sjöberg et al. [4]	32 adults, TBSA > 10%, ICU, Linköping Burn Center, Sweden	91% infection; 50% sepsis; 31% septic shock in the first week	Early sepsis < 14 d (mostly week 1)	Prospective single-center; Sepsis-3 criteria used; No prophylactic antibiotics; TBSA 34.8%, mortality 12%
Knuth et al. [5]	Retrospective single-institution cohort study, Canada	Not reported	Mean 13 d	Compared multiple definitions; Sepsis-3 criteria most reliable; Characterized by temporal biomarker changes pre- and post-sepsis
Yoon et al. [6]	1659 adults, burn ICU, South Korea	All patients met Sepsis-3 criteria for analysis	Mean 4.4 d (range 1 – 31 d)	Used SOFA ≥ 2; Predictors: procalcitonin, pH, platelets; Mean TBSA 37.9%, 42.7% inhalation injury

*ICU* intensive care unit, *SOFA* Sequential Organ Failure Assessment, *TBSA* total body surface area

Tedesco et al. [7] have provided an important retrospective dataset that underscores the burden of sepsis in burn care. Their work not only strengthens the evidence base but also illustrates the need for more refined prospective studies. The future of burn sepsis research lies in early detection, harmonised definitions, explicit antibiotic reporting, and the integration of biomarkers with AI-driven analytics to improve patient outcomes.

## Data Availability

Not applicable.

## Abbreviations

AI: Artificial intelligence
SOFA: Sequential Organ Failure Assessment
TBSA: Total body surface area
